# Supplementary material for: Morphological traits and machine learning for genetic lineage prediction of two reef-building corals
Source: PLoS One. 2025 Jun 18;20(6):e0326095. doi: 10.1371/journal.pone.0326095 (PMC12176140; doi:10.1371/journal.pone.0326095)
Supplement: S1 File — (DOCX) [file pone.0326095.s001.docx]

# S1 Appendix

## *Porites* spp.


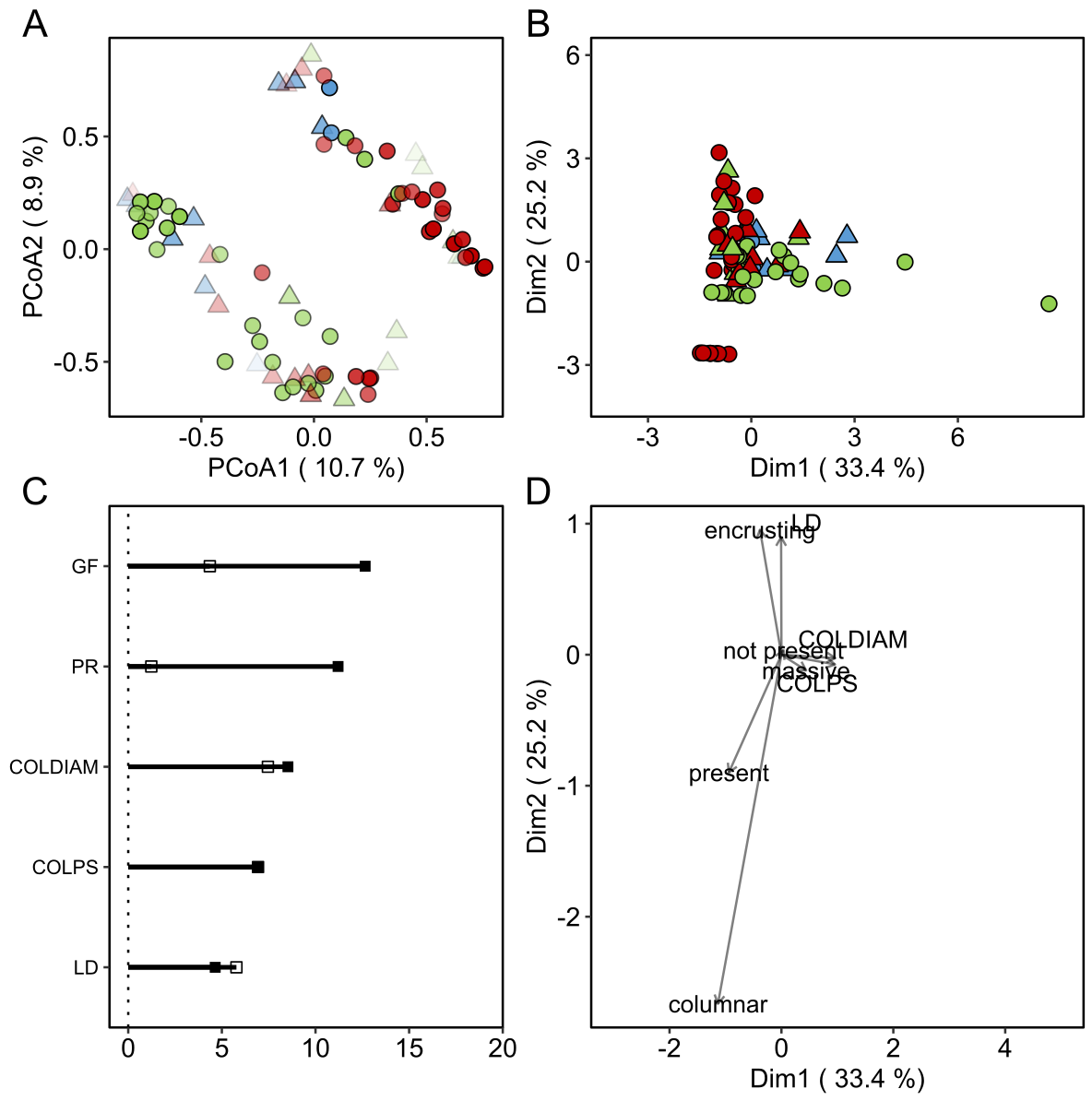
 **S1 Fig**. ***Porites* spp. corallum Random Forest model and Factor Analysis of Mixed Data**.

(A) Principal coordinate analysis of *Porites* corallum model proximity matrix. (B) Principal coordinate analysis of factor analysis of mixed data (FAMD) on corallum morphological annotations. Colors indicate the different lineages (see below), and the shapes indicate whether RF correctly or incorrectly classified the sample (circle: CC, triangle: WC). Confidence of prediction is represented in (A) as an alpha gradient (0-1). (C) *Porites* corallum model variables of importance; mean decrease accuracy and mean decrease Gini are shown as black and white squares, respectively. (D) FAMD loadings of quantitative and qualitative parameters. SSH1_pever is represented in blue, SSH2_plob in green, SSH3_plob in red.


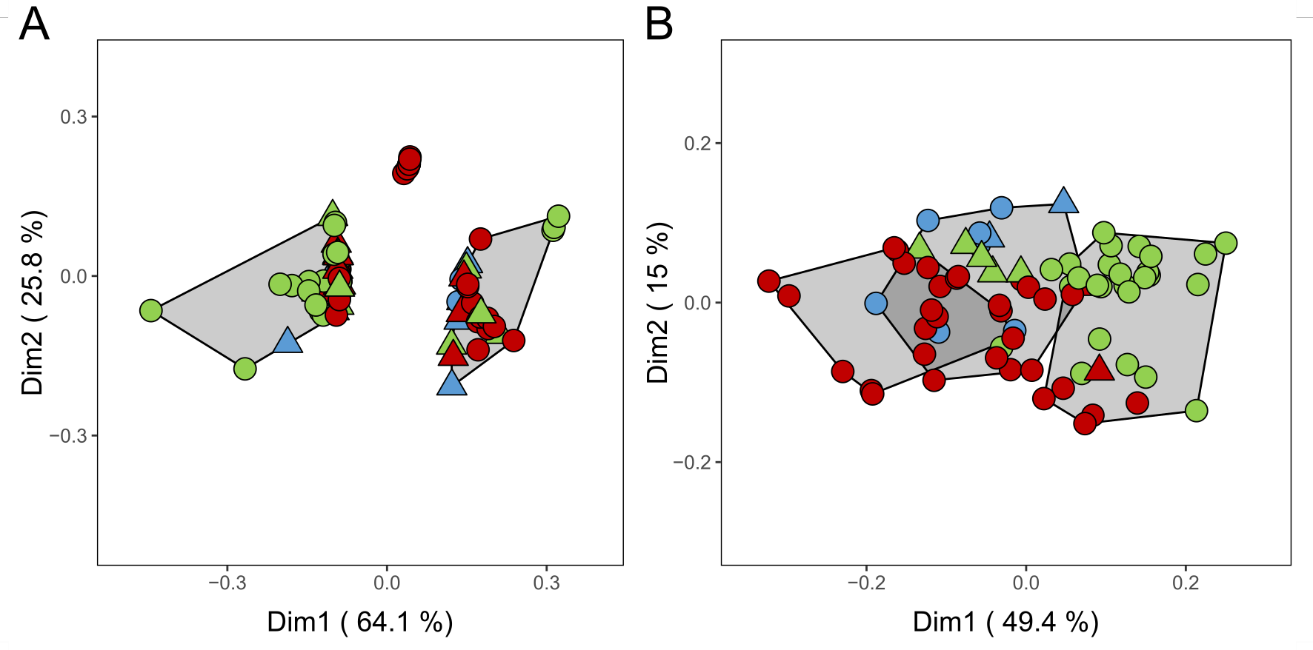


**S2 Fig. Principal coordinate analysis of *Porites* Gower distances.**

(A) Corallum morphology. (B) Corallum and corallite morphology. Colors indicate the different lineages (see below), and the shapes indicate whether RF correctly or incorrectly classified the sample (circle: CC, triangle: WC). Polygons show hierarchical clusters (k=3). SSH1_pever is represented in blue, SSH2_plob in green, SSH3_plob in red.

**S1 Table**. ***Porites* corallum pairwise PERMANOVA**

| **RF proximity matrix** | | | | | |
| --- | --- | --- | --- | --- | --- |
|  | **R2** | **F** | **df** | **p** | **p.adj** |
| SSH1_pever SSH2_plob | 0.05 | 2.26 | 1;44 | 0.024 | 0.024 |
| SSH1_pever SSH3_plob | 0.08 | 3.68 | 1;45 | 0.003 | 0.006 |
| SSH2_plob SSH3_plob | 0.05 | 3.67 | 1;67 | 0.001 | 0.003 |
| **Gower distance** | | | | | |
|  | **R2** | **F** | **df** | **p** | **p.adj** |
| SSH1*_*pever SSH2_plob | 0.03 | 1.48 | 1;44 | 0.243 | 0.243 |
| SSH1_pever SSH3_plob | 0.07 | 3.14 | 1;45 | 0.044 | 0.088 |
| SSH2_plob SSH3_plob | 0.09 | 6.56 | 1;67 | 0.002 | 0.006 |

**S2 Table**. ***Porites* corallum and corallite pairwise PERMANOVA**

| **RF proximity matrix** | | | | | |
| --- | --- | --- | --- | --- | --- |
|  | **R2** | **F** | **df** | **p** | **p.adj** |
| SSH1*_*pever SSH2_plob | 0.23 | 11.14 | 1;38 | 0.001 | 0.001 |
| SSH1_pever SSH3_plob | 0.20 | 10.56 | 1;41 | 0.001 | 0.001 |
| SSH2_plob SSH3_plob | 0.18 | 13.62 | 1;63 | 0.001 | 0.001 |
| **Gower distance** | | | | | |
|  | **R2** | **F** | **df** | **p** | **p.adj** |
| SSH1*_*pever SSH2_plob | 0.24 | 12.15 | 1;38 | 0.001 | 0.001 |
| SSH1_pever SSH3_plob | 0.13 | 6.09 | 1;41 | 0.001 | 0.001 |
| SSH2_plob SSH3_plob | 0.23 | 18.61 | 1;63 | 0.001 | 0.001 |

**S3 Table**. **Unit of measurement of *Porites* morphological parameters**

| **mm** | **mm^2^** | **count** | **count/mm^2^** |
| --- | --- | --- | --- |
| COLDIAM | COLPS | PR | LD |
| CAS | CAW | GF | - |
| SEPL | CAA | PN | - |
| SEPSVENA | FA | RN | - |
| SEPSVENS | COPS | - | - |
| DORL | - | - | - |
| VENL | - | - | - |
| FW | - | - | - |
| DDPW | - | - | - |
| VDPW |  |  |  |


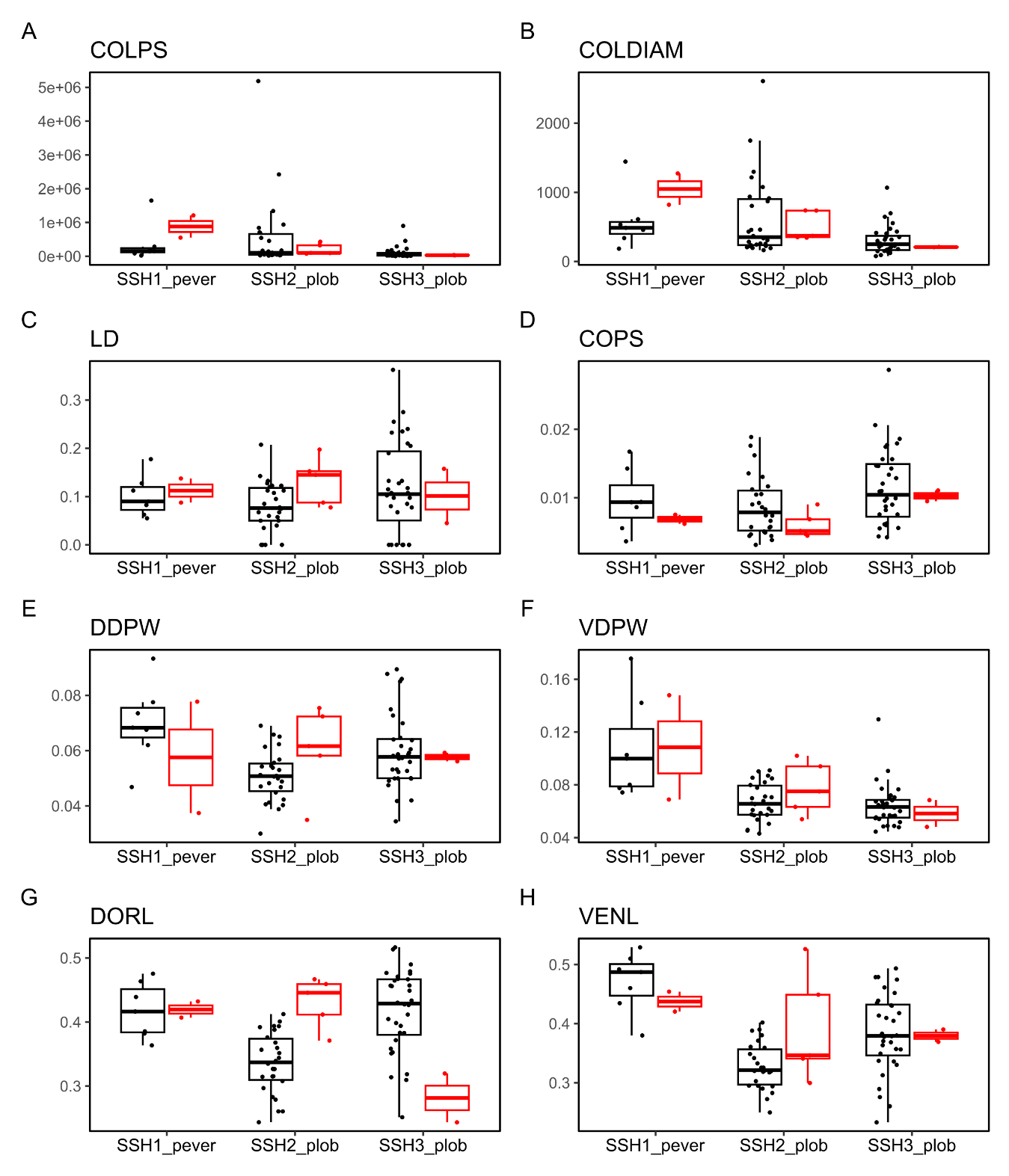


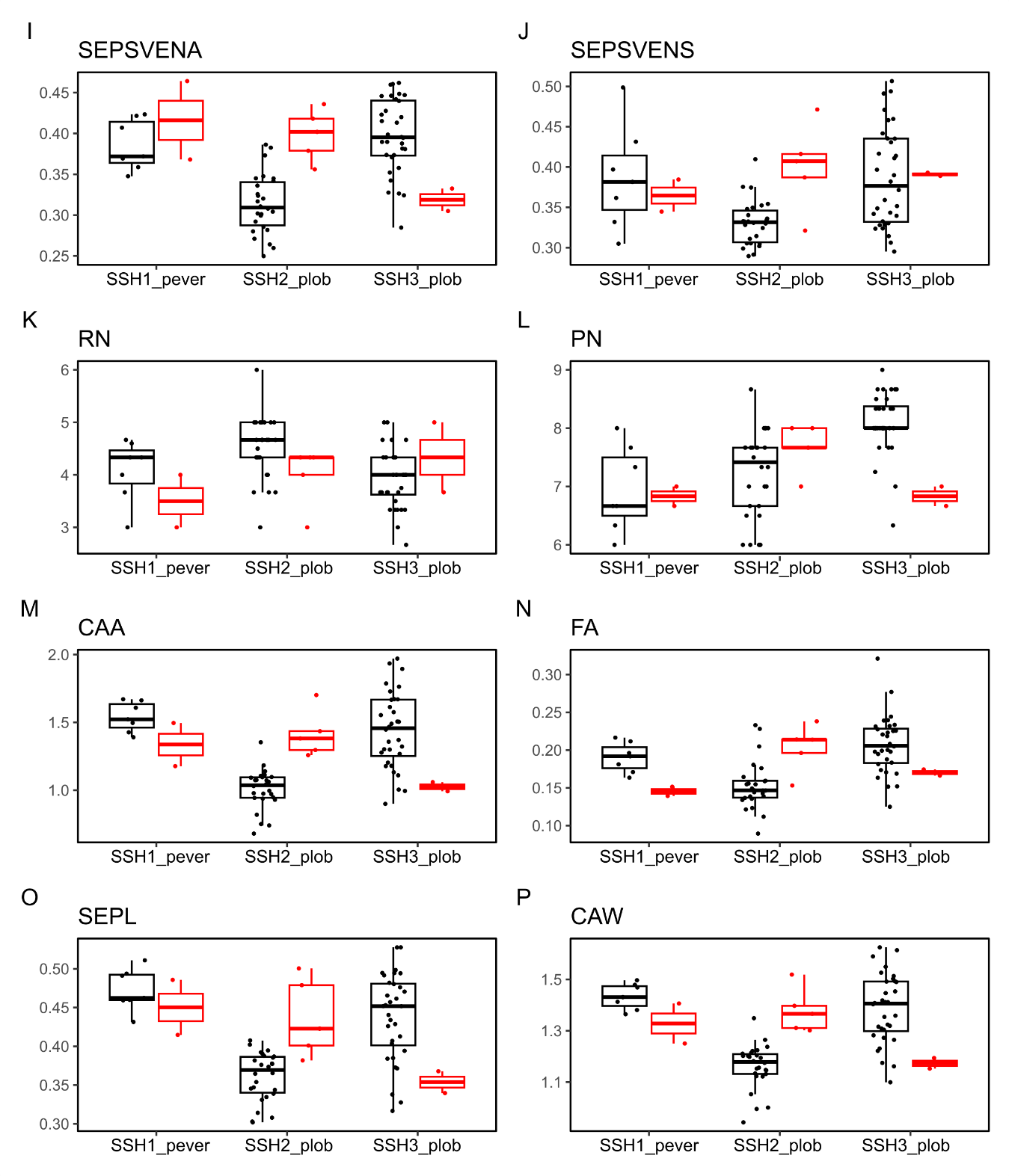


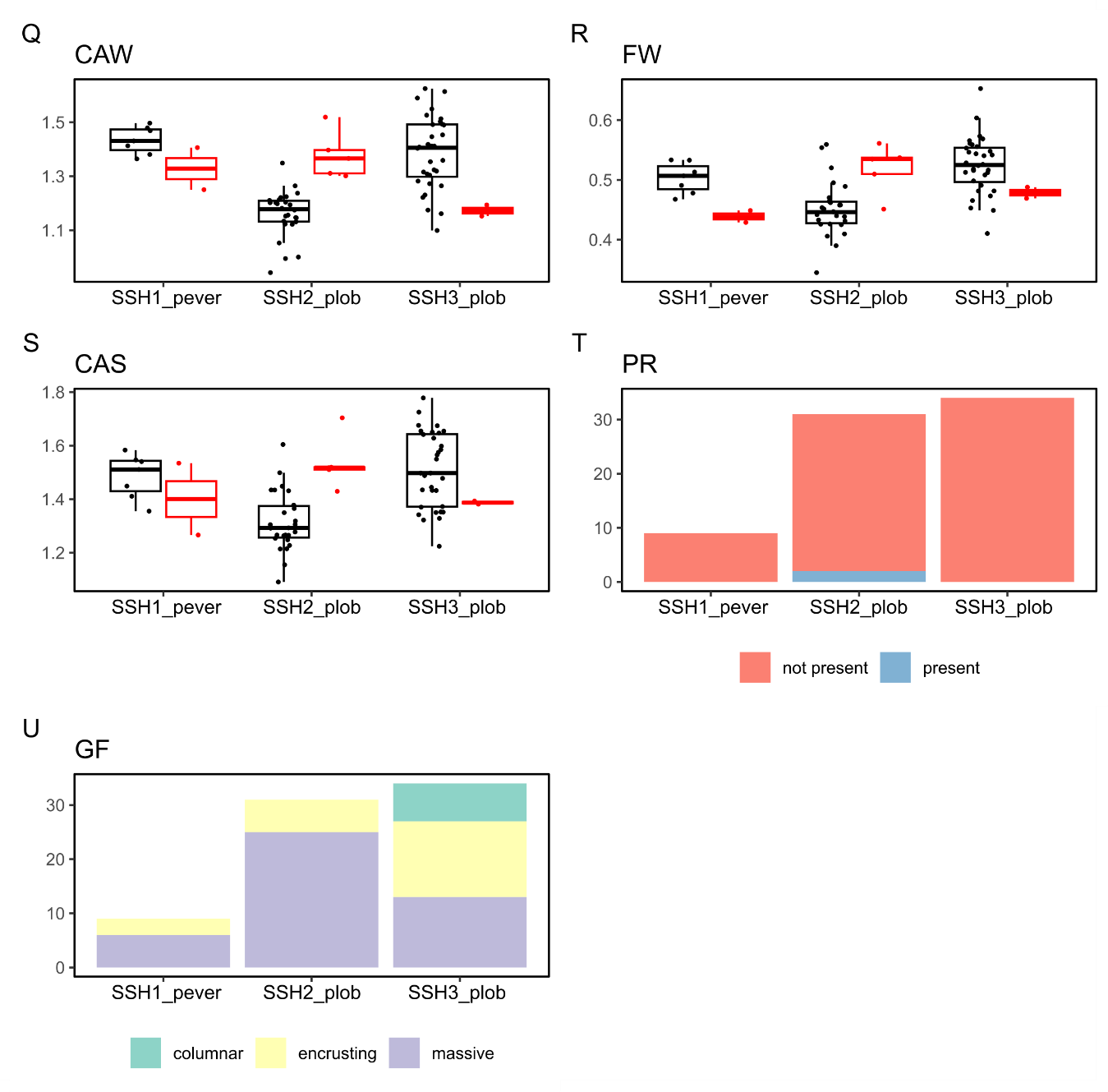


**S3 Fig. Univariate parameters of *Porites* morphology (A-U).**

Refer to Fig 1 and Table 1 in the main text for the full parameter’s description. Refer to supplementary materials, Table S3 for each plot measurement unit. Correctly classified corals (CC) by *Porites* corallum and corallite models in black, wrong classified corals (WC) in red.

## *Pocillopora* spp.


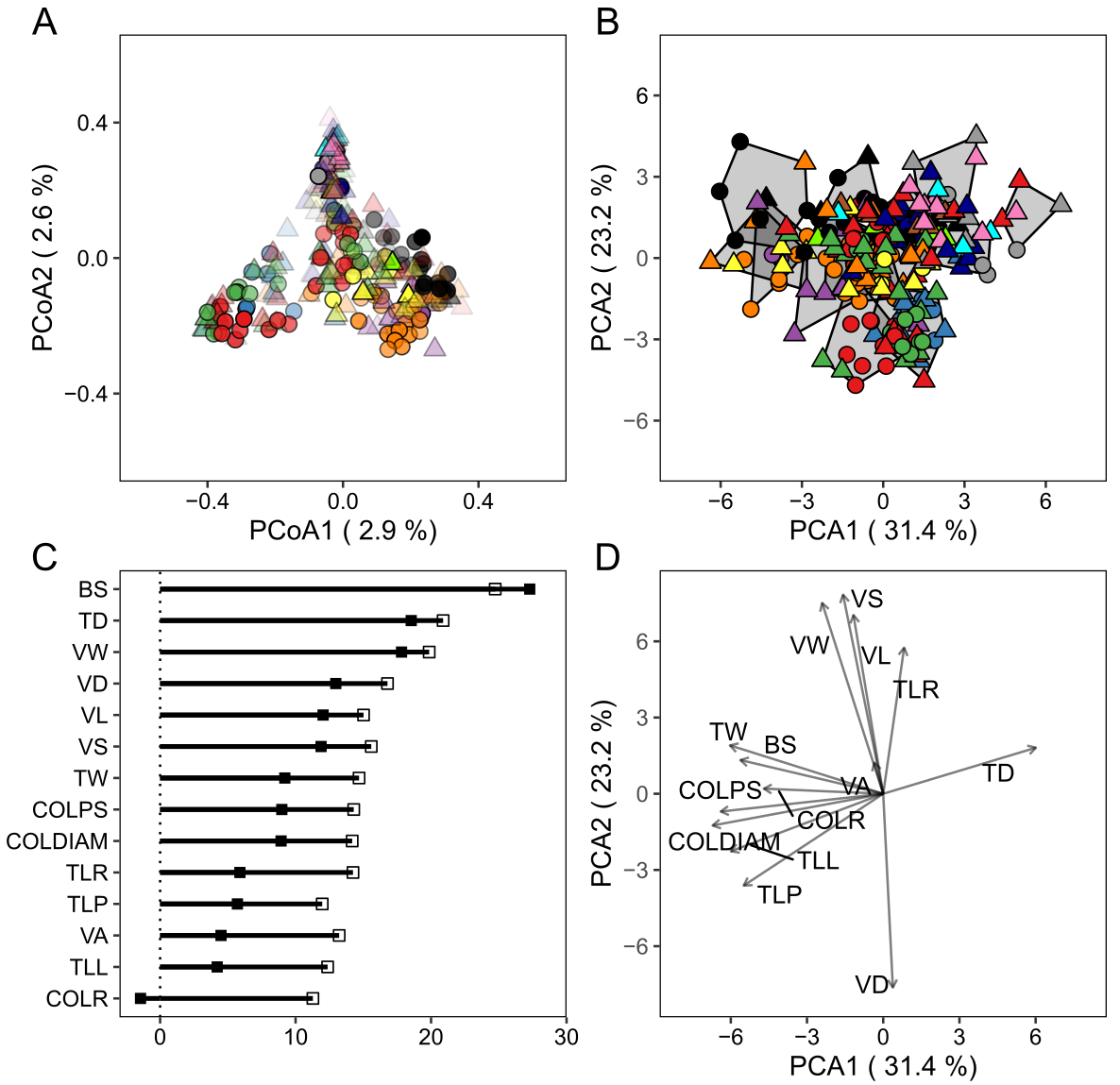
 **S4 Fig**. ***Pocillopora* spp. corallum and corallite Random Forest model and Principal component analysis.**

(A) Principal coordinate analysis of *Pocillopora* corallum model proximity matrix. (B) Principal component analysis (PCA) on corallum morphological annotations. Colors indicate the different lineages (see below) and the shapes indicate whether the RF model correctly or incorrectly classified the samples (circle: CC, triangle: WC). Polygons in (B) show k-means clusters (k=13). Confidence of prediction is represented in (A) as alpha gradient (0-1). (C) *Pocillopora* corallum model’s variables of importance; mean decrease accuracy and mean decrease Gini are shown as black and white squares, respectively. (D) PCA parameter loadings. GSH09c represented in orange, GSH14 in blue, GSH01 in purple, GSH09b in green, GSH09a in black, GSH13c in red, GSH13a in dark blue, GSH12 in light green, GSH13b in yellow, GSH15 in cyan, GSH05 in grey, GSH10 in brown, GSH04 in pink.

**S4 Table. *Pocillopora* corallum pairwise PERMANOVA**

| **Random Forest proximity matrix** | | | | | |
| --- | --- | --- | --- | --- | --- |
| **Comparison** | **R2** | **F** | **df** | **p** | **p.adj** |
| GSH09c.GSH14 | 0.06 | 3.35 | 1;54 | 0.001 | 0.032 |
| GSH09c.GSH01 | 0.03 | 1.53 | 1;53 | 0.007 | 0.189 |
| GSH09c.GSH09b | 0.04 | 3.41 | 1;74 | 0.001 | 0.032 |
| GSH09c.GSH13c | 0.04 | 3.44 | 1;81 | 0.001 | 0.032 |
| GSH09c.GSH09a | 0.05 | 3.36 | 1;63 | 0.001 | 0.032 |
| GSH09c.GSH13a | 0.05 | 2.97 | 1;54 | 0.001 | 0.032 |
| GSH09c.GSH13b | 0.03 | 1.79 | 1;54 | 0.001 | 0.032 |
| GSH09c.GSH05 | 0.06 | 3.19 | 1;48 | 0.001 | 0.032 |
| GSH09c.GSH12 | 0.03 | 1.23 | 1;41 | 0.079 | 0.738 |
| GSH09c.GSH10 | 0.04 | 1.94 | 1;43 | 0.001 | 0.032 |
| GSH09c.GSH04 | 0.06 | 3.04 | 1;47 | 0.001 | 0.032 |
| GSH09c.GSH15 | 0.04 | 1.64 | 1;41 | 0.001 | 0.032 |
| GSH14.GSH01 | 0.10 | 3.39 | 1;31 | 0.001 | 0.032 |
| GSH14.GSH09b | 0.03 | 1.85 | 1;52 | 0.001 | 0.032 |
| GSH14.GSH13c | 0.04 | 2.16 | 1;59 | 0.001 | 0.032 |
| GSH14.GSH09a | 0.10 | 4.32 | 1;41 | 0.001 | 0.032 |
| GSH14.GSH13a | 0.08 | 2.60 | 1;32 | 0.001 | 0.032 |
| GSH14.GSH13b | 0.08 | 2.64 | 1;32 | 0.001 | 0.032 |
| GSH14.GSH05 | 0.10 | 2.79 | 1;26 | 0.001 | 0.032 |
| GSH14.GSH12 | 0.08 | 1.68 | 1;19 | 0.002 | 0.060 |
| GSH14.GSH10 | 0.09 | 2.11 | 1;21 | 0.001 | 0.032 |
| GSH14.GSH04 | 0.10 | 2.87 | 1;25 | 0.001 | 0.032 |
| GSH14.GSH15 | 0.09 | 1.84 | 1;19 | 0.001 | 0.032 |
| GSH01.GSH09b | 0.06 | 3.15 | 1;51 | 0.001 | 0.032 |
| GSH01.GSH13c | 0.05 | 3.14 | 1;58 | 0.001 | 0.032 |
| GSH01.GSH09a | 0.08 | 3.39 | 1;40 | 0.001 | 0.032 |
| GSH01.GSH13a | 0.09 | 2.97 | 1;31 | 0.001 | 0.032 |
| GSH01.GSH13b | 0.05 | 1.76 | 1;31 | 0.001 | 0.032 |
| GSH01.GSH05 | 0.11 | 3.16 | 1;25 | 0.001 | 0.032 |
| GSH01.GSH12 | 0.08 | 1.47 | 1;18 | 0.018 | 0.396 |
| GSH01.GSH10 | 0.10 | 2.12 | 1;20 | 0.001 | 0.032 |
| GSH01.GSH04 | 0.11 | 3.06 | 1;24 | 0.001 | 0.032 |
| GSH01.GSH15 | 0.09 | 1.88 | 1;18 | 0.001 | 0.032 |
| GSH09b.GSH13c | 0.01 | 0.96 | 1;79 | 0.556 | 0.738 |
| GSH09b.GSH09a | 0.06 | 3.80 | 1;61 | 0.001 | 0.032 |
| GSH09b.GSH13a | 0.03 | 1.82 | 1;52 | 0.001 | 0.032 |
| GSH09b.GSH13b | 0.04 | 2.20 | 1;52 | 0.001 | 0.032 |
| GSH09b.GSH05 | 0.05 | 2.56 | 1;46 | 0.001 | 0.032 |
| GSH09b.GSH12 | 0.03 | 1.22 | 1;39 | 0.077 | 0.738 |
| GSH09b.GSH10 | 0.03 | 1.46 | 1;41 | 0.012 | 0.276 |
| GSH09b.GSH04 | 0.05 | 2.44 | 1;45 | 0.001 | 0.032 |
| GSH09b.GSH15 | 0.04 | 1.50 | 1;39 | 0.009 | 0.234 |
| GSH13c.GSH09a | 0.05 | 3.76 | 1;68 | 0.001 | 0.032 |
| GSH13c.GSH13a | 0.03 | 1.83 | 1;59 | 0.001 | 0.032 |
| GSH13c.GSH13b | 0.04 | 2.48 | 1;59 | 0.001 | 0.032 |
| GSH13c.GSH05 | 0.04 | 2.49 | 1;53 | 0.001 | 0.032 |
| GSH13c.GSH12 | 0.03 | 1.21 | 1;46 | 0.093 | 0.738 |
| GSH13c.GSH10 | 0.03 | 1.59 | 1;48 | 0.002 | 0.060 |
| GSH13c.GSH04 | 0.04 | 2.31 | 1;52 | 0.001 | 0.032 |
| GSH13c.GSH15 | 0.03 | 1.44 | 1;46 | 0.012 | 0.276 |
| GSH09a.GSH13a | 0.07 | 2.85 | 1;41 | 0.001 | 0.032 |
| GSH09a.GSH13b | 0.06 | 2.77 | 1;41 | 0.001 | 0.032 |
| GSH09a.GSH05 | 0.08 | 3.25 | 1;35 | 0.001 | 0.032 |
| GSH09a.GSH12 | 0.04 | 1.26 | 1;28 | 0.133 | 0.738 |
| GSH09a.GSH10 | 0.06 | 2.06 | 1;30 | 0.001 | 0.032 |
| GSH09a.GSH04 | 0.08 | 3.07 | 1;34 | 0.001 | 0.032 |
| GSH09a.GSH15 | 0.05 | 1.60 | 1;28 | 0.023 | 0.483 |
| GSH13a.GSH13b | 0.06 | 2.19 | 1;32 | 0.001 | 0.032 |
| GSH13a.GSH05 | 0.05 | 1.38 | 1;26 | 0.032 | 0.608 |
| GSH13a.GSH12 | 0.05 | 1.07 | 1;19 | 0.24 | 0.738 |
| GSH13a.GSH10 | 0.05 | 1.07 | 1;21 | 0.307 | 0.738 |
| GSH13a.GSH04 | 0.04 | 0.99 | 1;25 | 0.502 | 0.738 |
| GSH13a.GSH15 | 0.05 | 1.00 | 1;19 | 0.427 | 0.738 |
| GSH13b.GSH05 | 0.09 | 2.59 | 1;26 | 0.001 | 0.032 |
| GSH13b.GSH12 | 0.06 | 1.21 | 1;19 | 0.091 | 0.738 |
| GSH13b.GSH10 | 0.07 | 1.67 | 1;21 | 0.004 | 0.116 |
| GSH13b.GSH04 | 0.09 | 2.54 | 1;25 | 0.001 | 0.032 |
| GSH13b.GSH15 | 0.07 | 1.51 | 1;19 | 0.005 | 0.14 |
| GSH05.GSH12 | 0.08 | 1.15 | 1;13 | 0.216 | 0.738 |
| GSH05.GSH10 | 0.10 | 1.63 | 1;15 | 0.011 | 0.275 |
| GSH05.GSH04 | 0.04 | 0.89 | 1;19 | 0.639 | 0.738 |
| GSH05.GSH15 | 0.07 | 0.98 | 1;13 | 0.506 | 0.738 |
| GSH12.GSH10 | 0.11 | 0.96 | 1;8 | 0.483 | 0.738 |
| GSH12.GSH04 | 0.09 | 1.19 | 1;12 | 0.132 | 0.738 |
| GSH12.GSH15 | 0.13 | 0.87 | 1;6 | 0.738 | 0.738 |
| GSH10.GSH04 | 0.09 | 1.45 | 1;14 | 0.026 | 0.520 |
| GSH10.GSH15 | 0.14 | 1.32 | 1;8 | 0.078 | 0.738 |
| GSH04.GSH15 | 0.08 | 0.99 | 1;12 | 0.453 | 0.738 |
| **Euclidean distances** | | | | | |
| **Comparison** | **R2** | **F** | **df** | **p** | **p.adj** |
| GSH09c.GSH14 | 0.16 | 9.99 | 1;54 | 0.002 | 0.122 |
| GSH09c.GSH01 | 0.01 | 0.56 | 1;53 | 0.477 | 0.981 |
| GSH09c.GSH09b | 0.15 | 13.41 | 1;74 | 0.001 | 0.067 |
| GSH09c.GSH13c | 0.11 | 10.48 | 1;81 | 0.002 | 0.122 |
| GSH09c.GSH09a | 0.08 | 5.21 | 1;63 | 0.022 | 0.981 |
| GSH09c.GSH13a | 0.21 | 14.34 | 1;54 | 0.002 | 0.122 |
| GSH09c.GSH13b | 0.01 | 0.44 | 1;54 | 0.521 | 0.981 |
| GSH09c.GSH05 | 0.22 | 13.71 | 1;48 | 0.001 | 0.067 |
| GSH09c.GSH12 | 0.05 | 2.02 | 1;41 | 0.153 | 0.981 |
| GSH09c.GSH10 | 0.06 | 2.87 | 1;43 | 0.086 | 0.981 |
| GSH09c.GSH04 | 0.19 | 11.03 | 1;47 | 0.007 | 0.385 |
| GSH09c.GSH15 | 0.09 | 4.12 | 1;41 | 0.05 | 0.981 |
| GSH14.GSH01 | 0.32 | 14.92 | 1;31 | 0.001 | 0.067 |
| GSH14.GSH09b | 0.03 | 1.84 | 1;52 | 0.189 | 0.981 |
| GSH14.GSH13c | 0.07 | 4.63 | 1;59 | 0.027 | 0.981 |
| GSH14.GSH09a | 0.04 | 1.55 | 1;41 | 0.251 | 0.981 |
| GSH14.GSH13a | 0.08 | 2.75 | 1;32 | 0.107 | 0.981 |
| GSH14.GSH13b | 0.18 | 7.06 | 1;32 | 0.017 | 0.867 |
| GSH14.GSH05 | 0.37 | 15.08 | 1;26 | 0.001 | 0.067 |
| GSH14.GSH12 | <0.01 | 0.09 | 1;19 | 0.778 | 0.981 |
| GSH14.GSH10 | 0.01 | 0.23 | 1;21 | 0.64 | 0.981 |
| GSH14.GSH04 | 0.26 | 8.73 | 1;25 | 0.005 | 0.295 |
| GSH14.GSH15 | 0.13 | 2.78 | 1;19 | 0.1 | 0.981 |
| GSH01.GSH09b | 0.28 | 19.95 | 1;51 | 0.001 | 0.067 |
| GSH01.GSH13c | 0.22 | 16.20 | 1;58 | 0.001 | 0.067 |
| GSH01.GSH09a | 0.15 | 7.14 | 1;40 | 0.017 | 0.867 |
| GSH01.GSH13a | 0.39 | 19.67 | 1;31 | 0.001 | 0.067 |
| GSH01.GSH13b | 0.05 | 1.50 | 1;31 | 0.242 | 0.981 |
| GSH01.GSH05 | 0.42 | 18.07 | 1;25 | 0.001 | 0.067 |
| GSH01.GSH12 | 0.14 | 3.05 | 1;18 | 0.076 | 0.981 |
| GSH01.GSH10 | 0.18 | 4.39 | 1;20 | 0.06 | 0.981 |
| GSH01.GSH04 | 0.38 | 14.78 | 1;24 | 0.002 | 0.122 |
| GSH01.GSH15 | 0.23 | 5.50 | 1;18 | 0.027 | 0.981 |
| GSH09b.GSH13c | 0.02 | 1.30 | 1;79 | 0.26 | 0.981 |
| GSH09b.GSH09a | 0.01 | 0.53 | 1;61 | 0.459 | 0.981 |
| GSH09b.GSH13a | 0.13 | 8.06 | 1;52 | 0.006 | 0.342 |
| GSH09b.GSH13b | 0.12 | 7.09 | 1;52 | 0.007 | 0.385 |
| GSH09b.GSH05 | 0.27 | 16.64 | 1;46 | 0.001 | 0.067 |
| GSH09b.GSH12 | <0.01 | 0.16 | 1;39 | 0.682 | 0.981 |
| GSH09b.GSH10 | 0.00 | 0.15 | 1;41 | 0.712 | 0.981 |
| GSH09b.GSH04 | 0.20 | 11.21 | 1;45 | 0.004 | 0.240 |
| GSH09b.GSH15 | 0.09 | 3.83 | 1;39 | 0.064 | 0.981 |
| GSH13c.GSH09a | <0.01 | 0.00 | 1;68 | 0.981 | 0.981 |
| GSH13c.GSH13a | 0.17 | 12.22 | 1;59 | 0.001 | 0.067 |
| GSH13c.GSH13b | 0.07 | 4.31 | 1;59 | 0.046 | 0.981 |
| GSH13c.GSH05 | 0.27 | 19.24 | 1;53 | 0.001 | 0.067 |
| GSH13c.GSH12 | 0.01 | 0.67 | 1;46 | 0.434 | 0.981 |
| GSH13c.GSH10 | 0.02 | 0.80 | 1;48 | 0.381 | 0.981 |
| GSH13c.GSH04 | 0.21 | 13.73 | 1;52 | 0.002 | 0.122 |
| GSH13c.GSH15 | 0.10 | 4.84 | 1;46 | 0.035 | 0.981 |
| GSH09a.GSH13a | 0.09 | 4.09 | 1;41 | 0.052 | 0.981 |
| GSH09a.GSH13b | 0.04 | 1.80 | 1;41 | 0.179 | 0.981 |
| GSH09a.GSH05 | 0.15 | 6.03 | 1;35 | 0.013 | 0.689 |
| GSH09a.GSH12 | 0.01 | 0.22 | 1;28 | 0.668 | 0.981 |
| GSH09a.GSH10 | 0.01 | 0.27 | 1;30 | 0.598 | 0.981 |
| GSH09a.GSH04 | 0.11 | 4.32 | 1;34 | 0.056 | 0.981 |
| GSH09a.GSH15 | 0.05 | 1.51 | 1;28 | 0.215 | 0.981 |
| GSH13a.GSH13b | 0.26 | 11.08 | 1;32 | 0.002 | 0.122 |
| GSH13a.GSH05 | 0.13 | 3.73 | 1;26 | 0.049 | 0.981 |
| GSH13a.GSH12 | 0.06 | 1.21 | 1;19 | 0.275 | 0.981 |
| GSH13a.GSH10 | 0.09 | 2.03 | 1;21 | 0.138 | 0.981 |
| GSH13a.GSH04 | 0.05 | 1.40 | 1;25 | 0.252 | 0.981 |
| GSH13a.GSH15 | 0.02 | 0.31 | 1;19 | 0.571 | 0.981 |
| GSH13b.GSH05 | 0.32 | 12.01 | 1;26 | 0.001 | 0.067 |
| GSH13b.GSH12 | 0.07 | 1.34 | 1;19 | 0.286 | 0.981 |
| GSH13b.GSH10 | 0.08 | 1.86 | 1;21 | 0.206 | 0.981 |
| GSH13b.GSH04 | 0.27 | 9.39 | 1;25 | 0.008 | 0.432 |
| GSH13b.GSH15 | 0.15 | 3.42 | 1;19 | 0.062 | 0.981 |
| GSH05.GSH12 | 0.28 | 5.11 | 1;13 | 0.041 | 0.981 |
| GSH05.GSH10 | 0.33 | 7.55 | 1;15 | 0.006 | 0.342 |
| GSH05.GSH04 | 0.04 | 0.70 | 1;19 | 0.417 | 0.981 |
| GSH05.GSH15 | 0.05 | 0.67 | 1;13 | 0.482 | 0.981 |
| GSH12.GSH10 | <0.01 | 0.00 | 1;8 | 0.981 | 0.981 |
| GSH12.GSH04 | 0.21 | 3.11 | 1;12 | 0.091 | 0.981 |
| GSH12.GSH15 | 0.13 | 0.93 | 1;6 | 0.388 | 0.981 |
| GSH10.GSH04 | 0.25 | 4.71 | 1;14 | 0.021 | 0.981 |
| GSH10.GSH15 | 0.16 | 1.48 | 1;8 | 0.287 | 0.981 |
| GSH04.GSH15 | 0.01 | 0.06 | 1;12 | 0.822 | 0.981 |

**S5 Table. *Pocillopora* corallum and corallite pairwise PERMANOVA**

| **Random Forest proximity matrix** | | | | | |
| --- | --- | --- | --- | --- | --- |
| **Comparison** | **R2** | **F** | **df** | **p** | **p.adj** |
| GSH09c.GSH14 | 0.22 | 14.35 | 1;50 | 0.001 | 0.027 |
| GSH09c.GSH01 | 0.15 | 8.74 | 1;48 | 0.001 | 0.027 |
| GSH09c.GSH09b | 0.18 | 14.20 | 1;65 | 0.001 | 0.027 |
| GSH09c.GSH13c | 0.15 | 13.41 | 1;75 | 0.001 | 0.027 |
| GSH09c.GSH09a | 0.20 | 14.68 | 1;58 | 0.001 | 0.027 |
| GSH09c.GSH13a | 0.15 | 8.62 | 1;47 | 0.001 | 0.027 |
| GSH09c.GSH13b | 0.17 | 9.97 | 1;47 | 0.001 | 0.027 |
| GSH09c.GSH05 | 0.18 | 9.32 | 1;43 | 0.001 | 0.027 |
| GSH09c.GSH12 | 0.11 | 4.44 | 1;37 | 0.001 | 0.027 |
| GSH09c.GSH10 | 0.11 | 4.82 | 1;39 | 0.001 | 0.027 |
| GSH09c.GSH04 | 0.15 | 7.02 | 1;39 | 0.001 | 0.027 |
| GSH09c.GSH15 | 0.11 | 4.39 | 1;37 | 0.002 | 0.046 |
| GSH14.GSH01 | 0.25 | 10.21 | 1;30 | 0.001 | 0.027 |
| GSH14.GSH09b | 0.09 | 4.44 | 1;47 | 0.001 | 0.027 |
| GSH14.GSH13c | 0.07 | 4.49 | 1;57 | 0.001 | 0.027 |
| GSH14.GSH09a | 0.16 | 7.73 | 1;40 | 0.001 | 0.027 |
| GSH14.GSH13a | 0.13 | 4.24 | 1;29 | 0.001 | 0.027 |
| GSH14.GSH13b | 0.15 | 5.16 | 1;29 | 0.001 | 0.027 |
| GSH14.GSH05 | 0.17 | 5.19 | 1;25 | 0.001 | 0.027 |
| GSH14.GSH12 | 0.13 | 2.73 | 1;19 | 0.001 | 0.027 |
| GSH14.GSH10 | 0.13 | 3.05 | 1;21 | 0.002 | 0.046 |
| GSH14.GSH04 | 0.17 | 4.19 | 1;21 | 0.001 | 0.027 |
| GSH14.GSH15 | 0.17 | 3.99 | 1;19 | 0.001 | 0.027 |
| GSH01.GSH09b | 0.17 | 8.90 | 1;45 | 0.001 | 0.027 |
| GSH01.GSH13c | 0.13 | 8.35 | 1;55 | 0.001 | 0.027 |
| GSH01.GSH09a | 0.21 | 9.97 | 1;38 | 0.001 | 0.027 |
| GSH01.GSH13a | 0.19 | 6.44 | 1;27 | 0.001 | 0.027 |
| GSH01.GSH13b | 0.23 | 7.91 | 1;27 | 0.001 | 0.027 |
| GSH01.GSH05 | 0.25 | 7.61 | 1;23 | 0.001 | 0.027 |
| GSH01.GSH12 | 0.19 | 4.06 | 1;17 | 0.001 | 0.027 |
| GSH01.GSH10 | 0.20 | 4.62 | 1;19 | 0.001 | 0.027 |
| GSH01.GSH04 | 0.24 | 6.14 | 1;19 | 0.001 | 0.027 |
| GSH01.GSH15 | 0.23 | 5.03 | 1;17 | 0.001 | 0.027 |
| GSH09b.GSH13c | 0.02 | 1.67 | 1;72 | 0.005 | 0.090 |
| GSH09b.GSH09a | 0.09 | 5.59 | 1;55 | 0.001 | 0.027 |
| GSH09b.GSH13a | 0.04 | 2.07 | 1;44 | 0.001 | 0.027 |
| GSH09b.GSH13b | 0.07 | 3.43 | 1;44 | 0.001 | 0.027 |
| GSH09b.GSH05 | 0.09 | 3.75 | 1;40 | 0.001 | 0.027 |
| GSH09b.GSH12 | 0.04 | 1.56 | 1;34 | 0.011 | 0.154 |
| GSH09b.GSH10 | 0.04 | 1.57 | 1;36 | 0.009 | 0.135 |
| GSH09b.GSH04 | 0.07 | 2.65 | 1;36 | 0.001 | 0.027 |
| GSH09b.GSH15 | 0.08 | 2.84 | 1;34 | 0.001 | 0.027 |
| GSH13c.GSH09a | 0.07 | 5.13 | 1;65 | 0.001 | 0.027 |
| GSH13c.GSH13a | 0.03 | 1.88 | 1;54 | 0.001 | 0.027 |
| GSH13c.GSH13b | 0.06 | 3.51 | 1;54 | 0.001 | 0.027 |
| GSH13c.GSH05 | 0.06 | 3.30 | 1;50 | 0.001 | 0.027 |
| GSH13c.GSH12 | 0.03 | 1.36 | 1;44 | 0.039 | 0.312 |
| GSH13c.GSH10 | 0.04 | 1.67 | 1;46 | 0.003 | 0.060 |
| GSH13c.GSH04 | 0.05 | 2.45 | 1;46 | 0.001 | 0.027 |
| GSH13c.GSH15 | 0.05 | 2.47 | 1;44 | 0.001 | 0.027 |
| GSH09a.GSH13a | 0.09 | 3.53 | 1;37 | 0.001 | 0.027 |
| GSH09a.GSH13b | 0.09 | 3.57 | 1;37 | 0.001 | 0.027 |
| GSH09a.GSH05 | 0.13 | 4.94 | 1;33 | 0.001 | 0.027 |
| GSH09a.GSH12 | 0.04 | 1.18 | 1;27 | 0.194 | 0.637 |
| GSH09a.GSH10 | 0.07 | 2.16 | 1;29 | 0.002 | 0.046 |
| GSH09a.GSH04 | 0.11 | 3.57 | 1;29 | 0.001 | 0.027 |
| GSH09a.GSH15 | 0.11 | 3.28 | 1;27 | 0.001 | 0.027 |
| GSH13a.GSH13b | 0.09 | 2.58 | 1;26 | 0.001 | 0.027 |
| GSH13a.GSH05 | 0.07 | 1.63 | 1;22 | 0.014 | 0.182 |
| GSH13a.GSH12 | 0.06 | 1.03 | 1;16 | 0.386 | 0.637 |
| GSH13a.GSH10 | 0.05 | 1.05 | 1;18 | 0.36 | 0.637 |
| GSH13a.GSH04 | 0.07 | 1.36 | 1;18 | 0.026 | 0.26 |
| GSH13a.GSH15 | 0.12 | 2.20 | 1;16 | 0.003 | 0.060 |
| GSH13b.GSH05 | 0.14 | 3.52 | 1;22 | 0.001 | 0.027 |
| GSH13b.GSH12 | 0.09 | 1.68 | 1;16 | 0.003 | 0.060 |
| GSH13b.GSH10 | 0.09 | 1.86 | 1;18 | 0.002 | 0.046 |
| GSH13b.GSH04 | 0.14 | 2.97 | 1;18 | 0.001 | 0.027 |
| GSH13b.GSH15 | 0.16 | 2.96 | 1;16 | 0.001 | 0.027 |
| GSH05.GSH12 | 0.11 | 1.51 | 1;12 | 0.023 | 0.260 |
| GSH05.GSH10 | 0.12 | 1.93 | 1;14 | 0.006 | 0.096 |
| GSH05.GSH04 | 0.08 | 1.29 | 1;14 | 0.144 | 0.637 |
| GSH05.GSH15 | 0.19 | 2.88 | 1;12 | 0.006 | 0.096 |
| GSH12.GSH10 | 0.10 | 0.93 | 1;8 | 0.637 | 0.637 |
| GSH12.GSH04 | 0.14 | 1.30 | 1;8 | 0.107 | 0.637 |
| GSH12.GSH15 | 0.25 | 1.97 | 1;6 | 0.087 | 0.609 |
| GSH10.GSH04 | 0.12 | 1.43 | 1;10 | 0.029 | 0.261 |
| GSH10.GSH15 | 0.19 | 1.92 | 1;8 | 0.024 | 0.260 |
| GSH04.GSH15 | 0.26 | 2.82 | 1;8 | 0.005 | 0.090 |
| **Euclidean distances** | | | | | |
| **Comparison** | **R2** | **F** | **df** | **p** | **p.adj** |
| GSH09c.GSH14 | 0.16 | 9.27 | 1;50 | 0.005 | 0.320 |
| GSH09c.GSH01 | 0.02 | 1.08 | 1;48 | 0.319 | 0.990 |
| GSH09c.GSH09b | 0.15 | 11.25 | 1;65 | 0.004 | 0.260 |
| GSH09c.GSH13c | 0.11 | 9.18 | 1;75 | 0.003 | 0.198 |
| GSH09c.GSH09a | 0.07 | 4.17 | 1;58 | 0.038 | 0.990 |
| GSH09c.GSH13a | 0.19 | 10.86 | 1;47 | 0.001 | 0.073 |
| GSH09c.GSH13b | 0.00 | 0.02 | 1;47 | 0.887 | 0.990 |
| GSH09c.GSH05 | 0.22 | 12.12 | 1;43 | 0.002 | 0.136 |
| GSH09c.GSH12 | 0.05 | 1.86 | 1;37 | 0.146 | 0.990 |
| GSH09c.GSH10 | 0.06 | 2.63 | 1;39 | 0.113 | 0.990 |
| GSH09c.GSH04 | 0.13 | 5.84 | 1;39 | 0.015 | 0.840 |
| GSH09c.GSH15 | 0.10 | 3.93 | 1;37 | 0.048 | 0.990 |
| GSH14.GSH01 | 0.35 | 16.32 | 1;30 | 0.001 | 0.073 |
| GSH14.GSH09b | 0.02 | 1.14 | 1;47 | 0.292 | 0.990 |
| GSH14.GSH13c | 0.07 | 4.09 | 1;57 | 0.051 | 0.990 |
| GSH14.GSH09a | 0.04 | 1.55 | 1;40 | 0.229 | 0.990 |
| GSH14.GSH13a | 0.06 | 1.98 | 1;29 | 0.153 | 0.990 |
| GSH14.GSH13b | 0.23 | 8.44 | 1;29 | 0.006 | 0.372 |
| GSH14.GSH05 | 0.36 | 14.28 | 1;25 | 0.002 | 0.136 |
| GSH14.GSH12 | <0.01 | 0.09 | 1;19 | 0.77 | 0.990 |
| GSH14.GSH10 | 0.01 | 0.23 | 1;21 | 0.631 | 0.990 |
| GSH14.GSH04 | 0.16 | 3.98 | 1;21 | 0.056 | 0.990 |
| GSH14.GSH15 | 0.13 | 2.78 | 1;19 | 0.095 | 0.990 |
| GSH01.GSH09b | 0.31 | 20.16 | 1;45 | 0.002 | 0.136 |
| GSH01.GSH13c | 0.25 | 18.04 | 1;55 | 0.002 | 0.136 |
| GSH01.GSH09a | 0.17 | 7.52 | 1;38 | 0.009 | 0.540 |
| GSH01.GSH13a | 0.39 | 17.05 | 1;27 | 0.001 | 0.073 |
| GSH01.GSH13b | 0.03 | 0.98 | 1;27 | 0.3 | 0.990 |
| GSH01.GSH05 | 0.43 | 17.61 | 1;23 | 0.001 | 0.073 |
| GSH01.GSH12 | 0.16 | 3.34 | 1;17 | 0.076 | 0.990 |
| GSH01.GSH10 | 0.20 | 4.83 | 1;19 | 0.038 | 0.990 |
| GSH01.GSH04 | 0.32 | 8.82 | 1;19 | 0.012 | 0.696 |
| GSH01.GSH15 | 0.26 | 5.87 | 1;17 | 0.022 | 0.990 |
| GSH09b.GSH13c | 0.02 | 1.34 | 1;72 | 0.244 | 0.990 |
| GSH09b.GSH09a | 0.01 | 0.71 | 1;55 | 0.429 | 0.990 |
| GSH09b.GSH13a | 0.10 | 4.67 | 1;44 | 0.03 | 0.990 |
| GSH09b.GSH13b | 0.16 | 8.64 | 1;44 | 0.006 | 0.372 |
| GSH09b.GSH05 | 0.24 | 12.73 | 1;40 | 0.001 | 0.073 |
| GSH09b.GSH12 | <0.01 | 0.08 | 1;34 | 0.806 | 0.990 |
| GSH09b.GSH10 | <0.01 | 0.05 | 1;36 | 0.838 | 0.990 |
| GSH09b.GSH04 | 0.11 | 4.42 | 1;36 | 0.045 | 0.990 |
| GSH09b.GSH15 | 0.08 | 3.04 | 1;34 | 0.089 | 0.990 |
| GSH13c.GSH09a | <0.01 | 0.02 | 1;65 | 0.883 | 0.990 |
| GSH13c.GSH13a | 0.14 | 8.78 | 1;54 | 0.002 | 0.136 |
| GSH13c.GSH13b | 0.10 | 6.26 | 1;54 | 0.014 | 0.798 |
| GSH13c.GSH05 | 0.25 | 16.92 | 1;50 | 0.001 | 0.073 |
| GSH13c.GSH12 | 0.01 | 0.57 | 1;44 | 0.448 | 0.990 |
| GSH13c.GSH10 | 0.01 | 0.67 | 1;46 | 0.436 | 0.990 |
| GSH13c.GSH04 | 0.13 | 6.63 | 1;46 | 0.012 | 0.696 |
| GSH13c.GSH15 | 0.09 | 4.53 | 1;44 | 0.031 | 0.990 |
| GSH09a.GSH13a | 0.08 | 3.14 | 1;37 | 0.078 | 0.990 |
| GSH09a.GSH13b | 0.06 | 2.38 | 1;37 | 0.129 | 0.990 |
| GSH09a.GSH05 | 0.14 | 5.47 | 1;33 | 0.019 | 0.990 |
| GSH09a.GSH12 | 0.01 | 0.22 | 1;27 | 0.666 | 0.990 |
| GSH09a.GSH10 | 0.01 | 0.27 | 1;29 | 0.607 | 0.990 |
| GSH09a.GSH04 | 0.07 | 2.17 | 1;29 | 0.159 | 0.990 |
| GSH09a.GSH15 | 0.05 | 1.48 | 1;27 | 0.233 | 0.990 |
| GSH13a.GSH13b | 0.28 | 9.95 | 1;26 | 0.007 | 0.427 |
| GSH13a.GSH05 | 0.13 | 3.40 | 1;22 | 0.062 | 0.990 |
| GSH13a.GSH12 | 0.05 | 0.90 | 1;16 | 0.346 | 0.990 |
| GSH13a.GSH10 | 0.08 | 1.52 | 1;18 | 0.242 | 0.990 |
| GSH13a.GSH04 | 0.02 | 0.43 | 1;18 | 0.577 | 0.990 |
| GSH13a.GSH15 | 0.02 | 0.32 | 1;16 | 0.559 | 0.990 |
| GSH13b.GSH05 | 0.35 | 11.85 | 1;22 | 0.003 | 0.198 |
| GSH13b.GSH12 | 0.09 | 1.63 | 1;16 | 0.246 | 0.990 |
| GSH13b.GSH10 | 0.11 | 2.30 | 1;18 | 0.135 | 0.990 |
| GSH13b.GSH04 | 0.24 | 5.58 | 1;18 | 0.023 | 0.990 |
| GSH13b.GSH15 | 0.19 | 3.72 | 1;16 | 0.051 | 0.990 |
| GSH05.GSH12 | 0.28 | 4.78 | 1;12 | 0.037 | 0.990 |
| GSH05.GSH10 | 0.34 | 7.06 | 1;14 | 0.019 | 0.990 |
| GSH05.GSH04 | 0.08 | 1.15 | 1;14 | 0.285 | 0.990 |
| GSH05.GSH15 | 0.05 | 0.69 | 1;12 | 0.474 | 0.990 |
| GSH12.GSH10 | <0.01 | 0.00 | 1;8 | 0.981 | 0.990 |
| GSH12.GSH04 | 0.15 | 1.42 | 1;8 | 0.289 | 0.990 |
| GSH12.GSH15 | 0.13 | 0.93 | 1;6 | 0.37 | 0.990 |
| GSH10.GSH04 | 0.18 | 2.21 | 1;10 | 0.17 | 0.990 |
| GSH10.GSH15 | 0.16 | 1.48 | 1;8 | 0.274 | 0.990 |
| GSH04.GSH15 | <0.01 | 0.00 | 1;8 | 0.99 | 0.990 |

**S6 Table. Unit of measurement of *Pocillopora* morphological parameters**

| **mm** | **mm^2^** | **count** | **count/mm^2^** | **degrees (^o^)** |
| --- | --- | --- | --- | --- |
| COLDIAM | COLPS | SEPN | LD | VA |
| TD | COPS | - | VD | - |
| TLL | - | - | - | - |
| TLP | - | - | - | - |
| TW | - | - | - | - |
| VS | - | - | - | - |
| VL | - | - | - | - |
| VW | - | - | - | - |
| CAS | - | - | - | - |
| CADIAM1 | - | - | - | - |
| CADIAM2 | - | - | - | - |
| SEPL | - | - | - | - |
| CODIAMF | - | - | - | - |
| SPIS | - | - | - | - |
| SPIW | - | - | - | - |


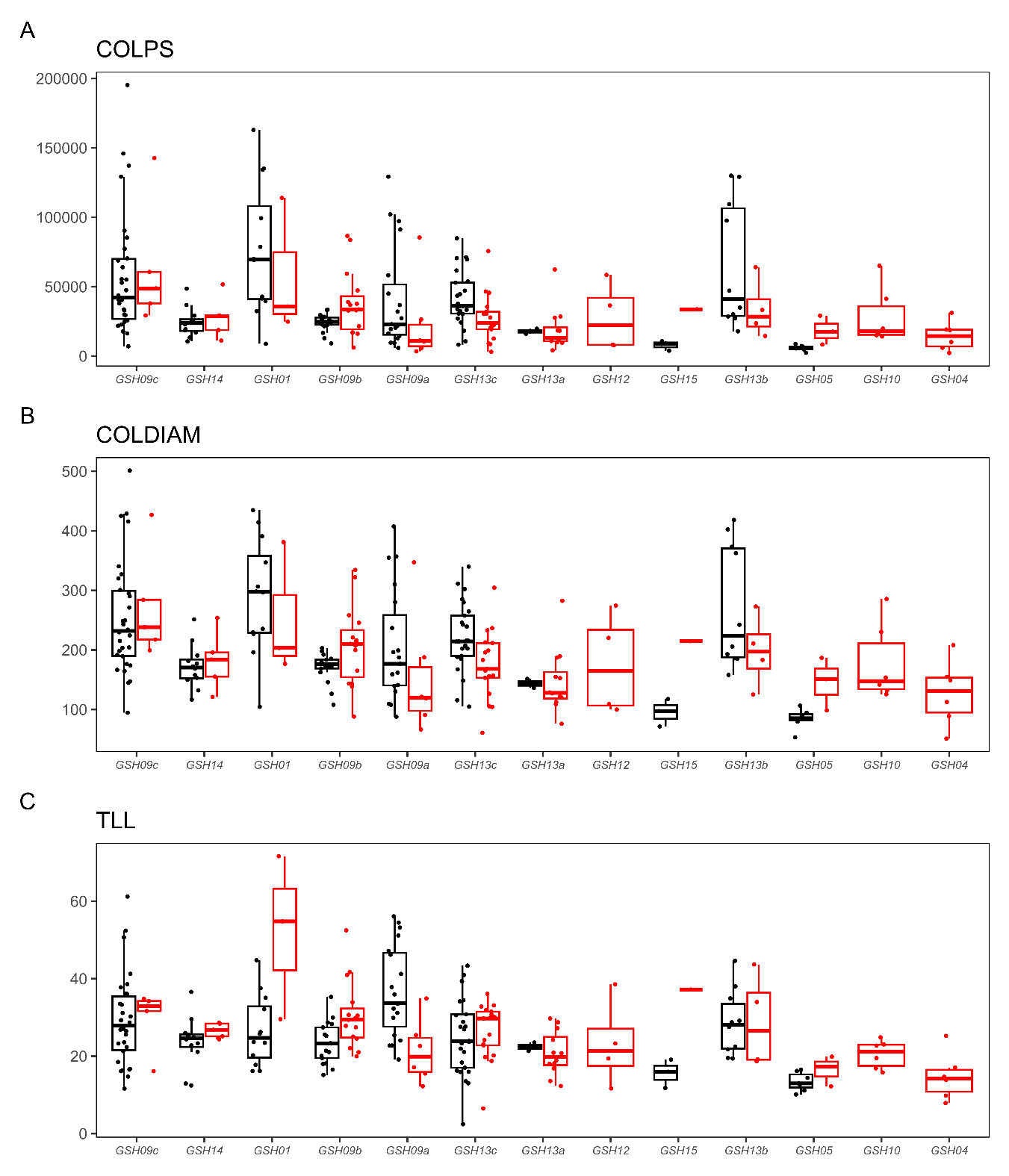


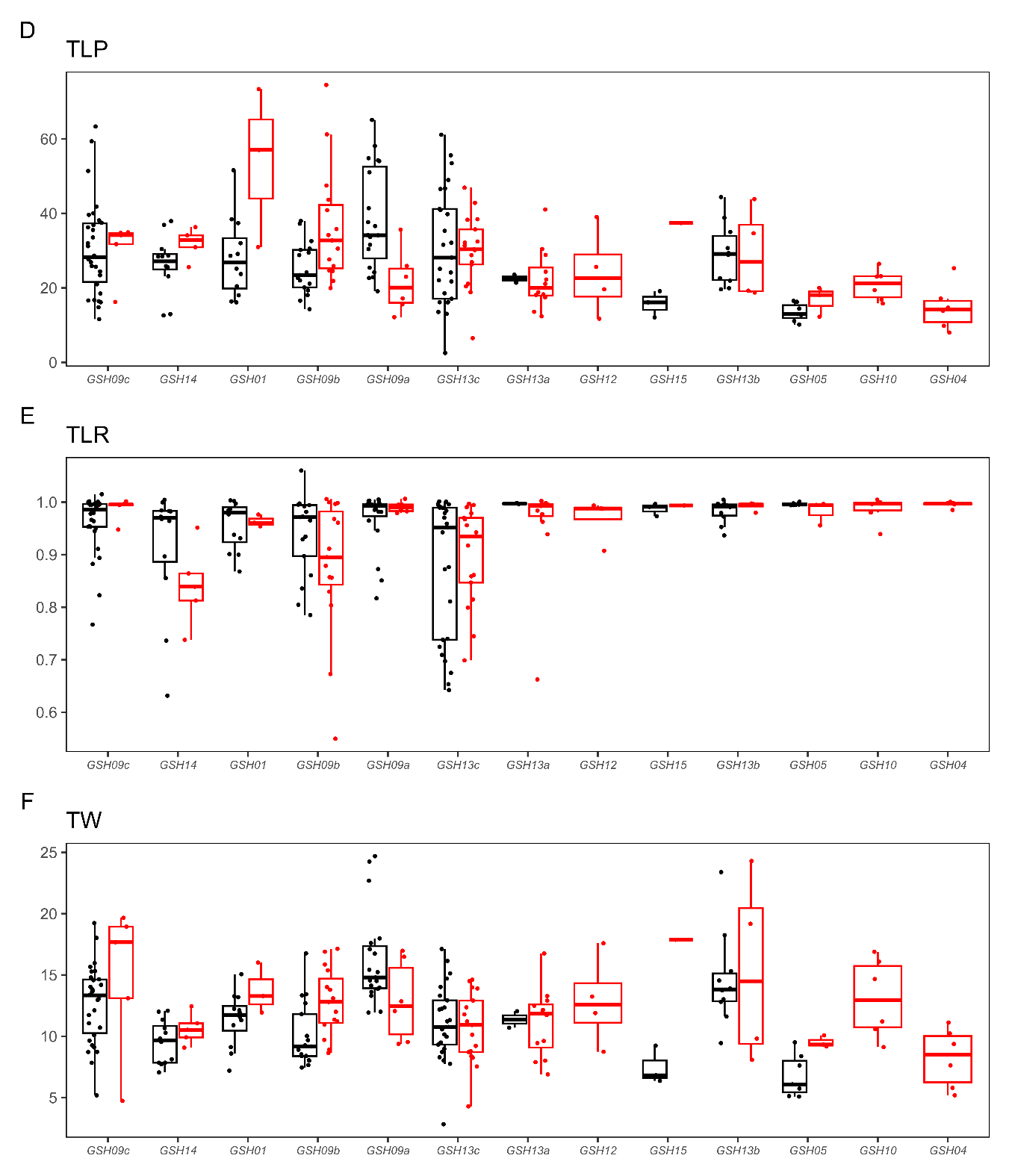


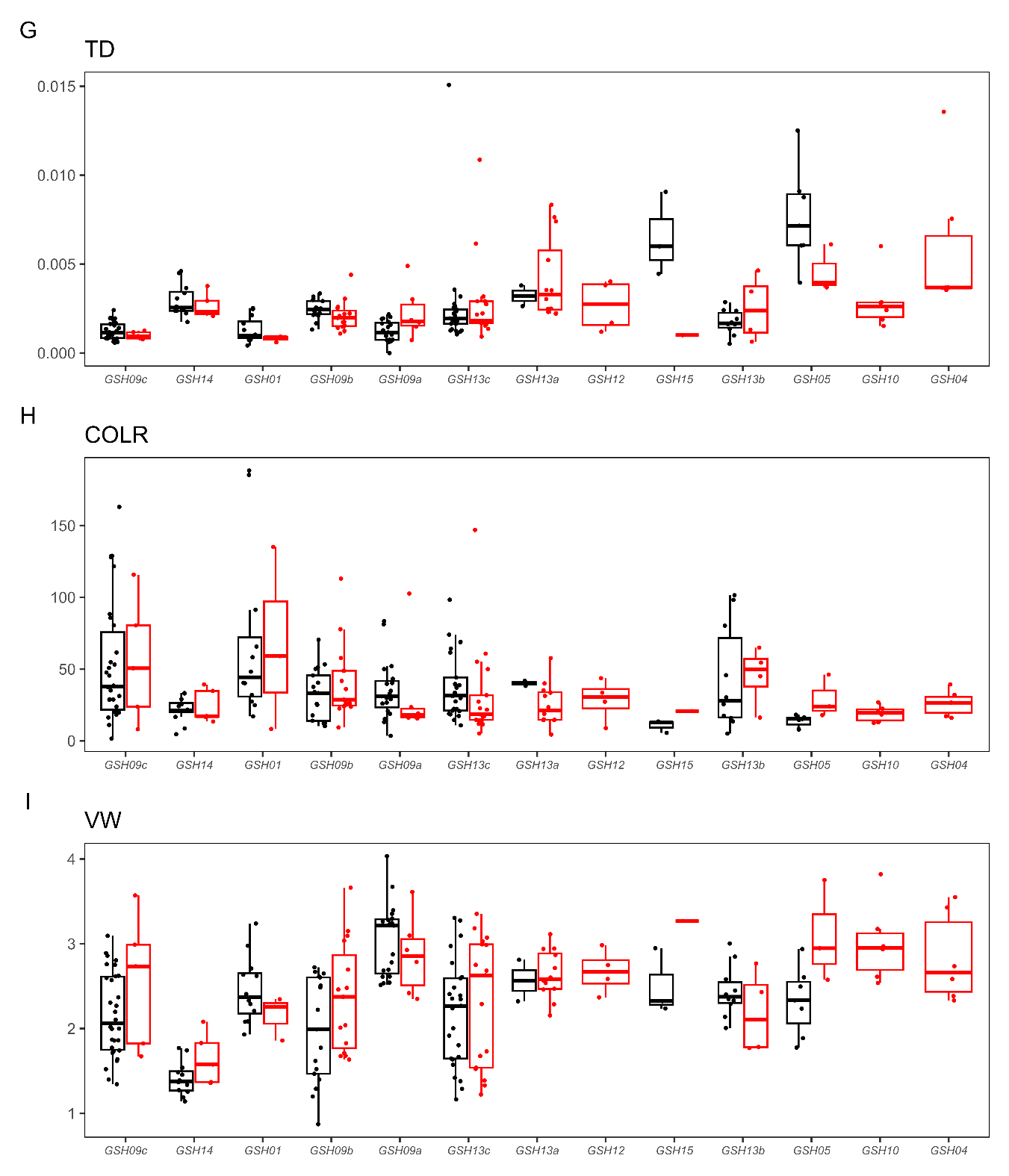


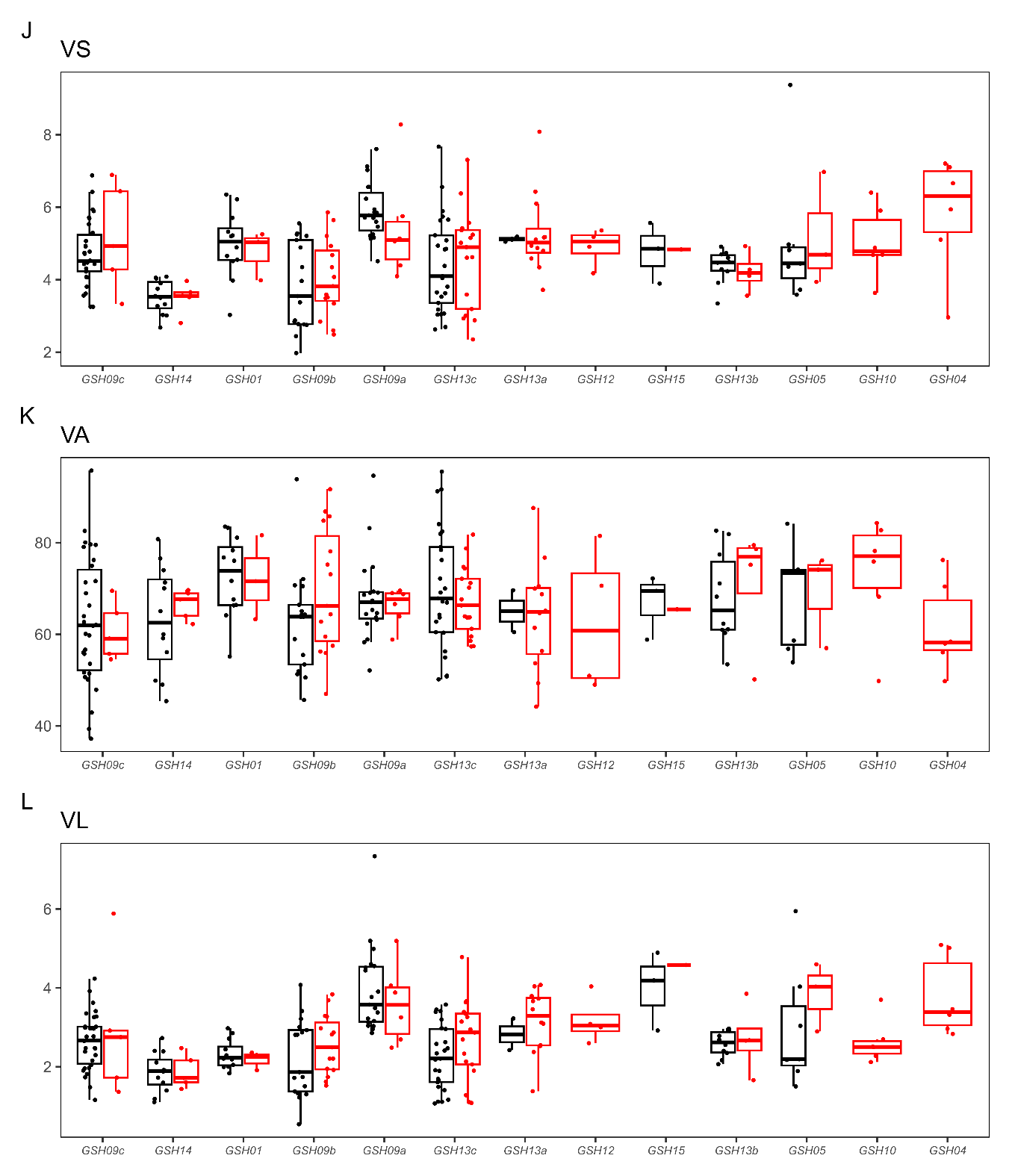


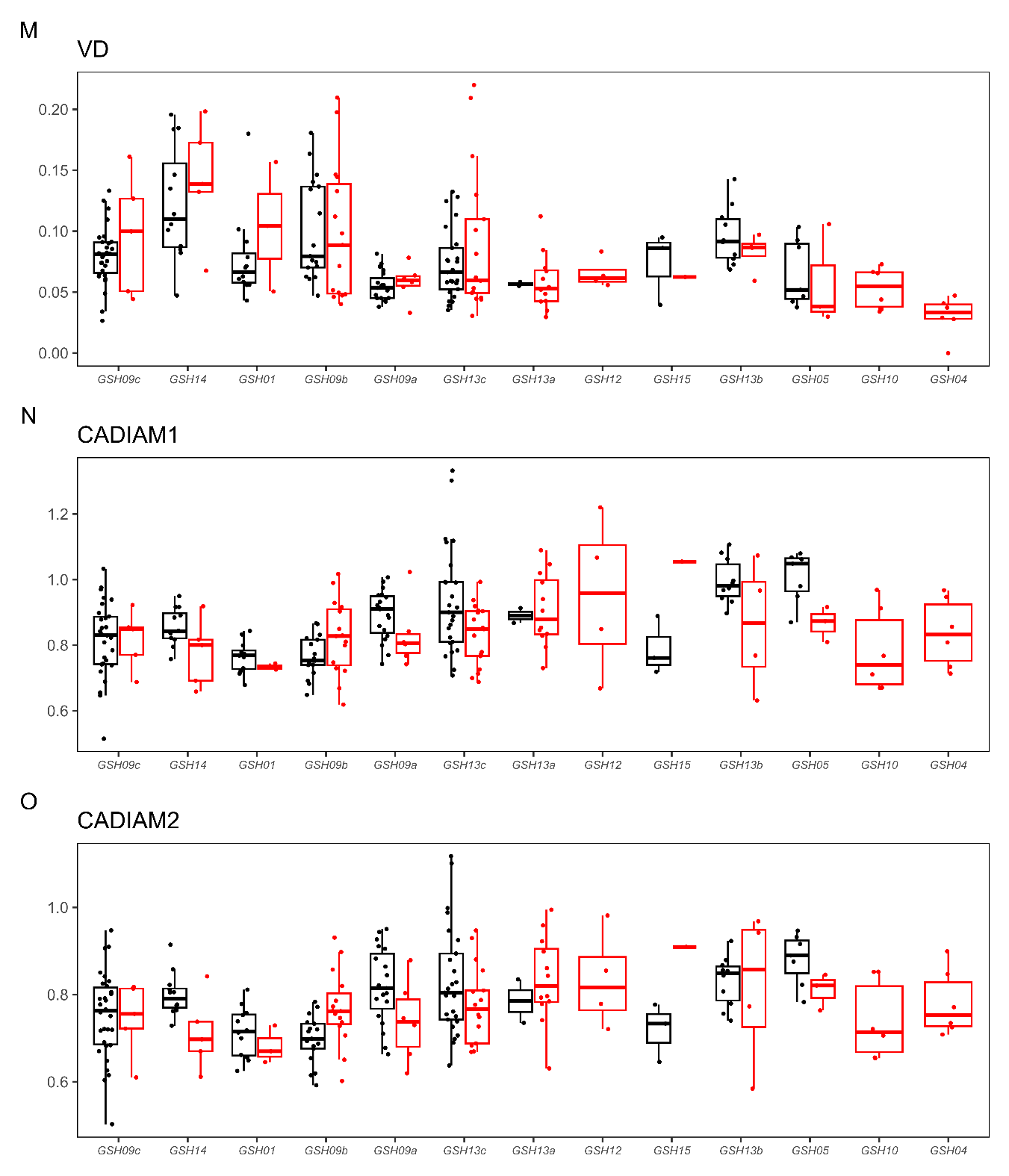


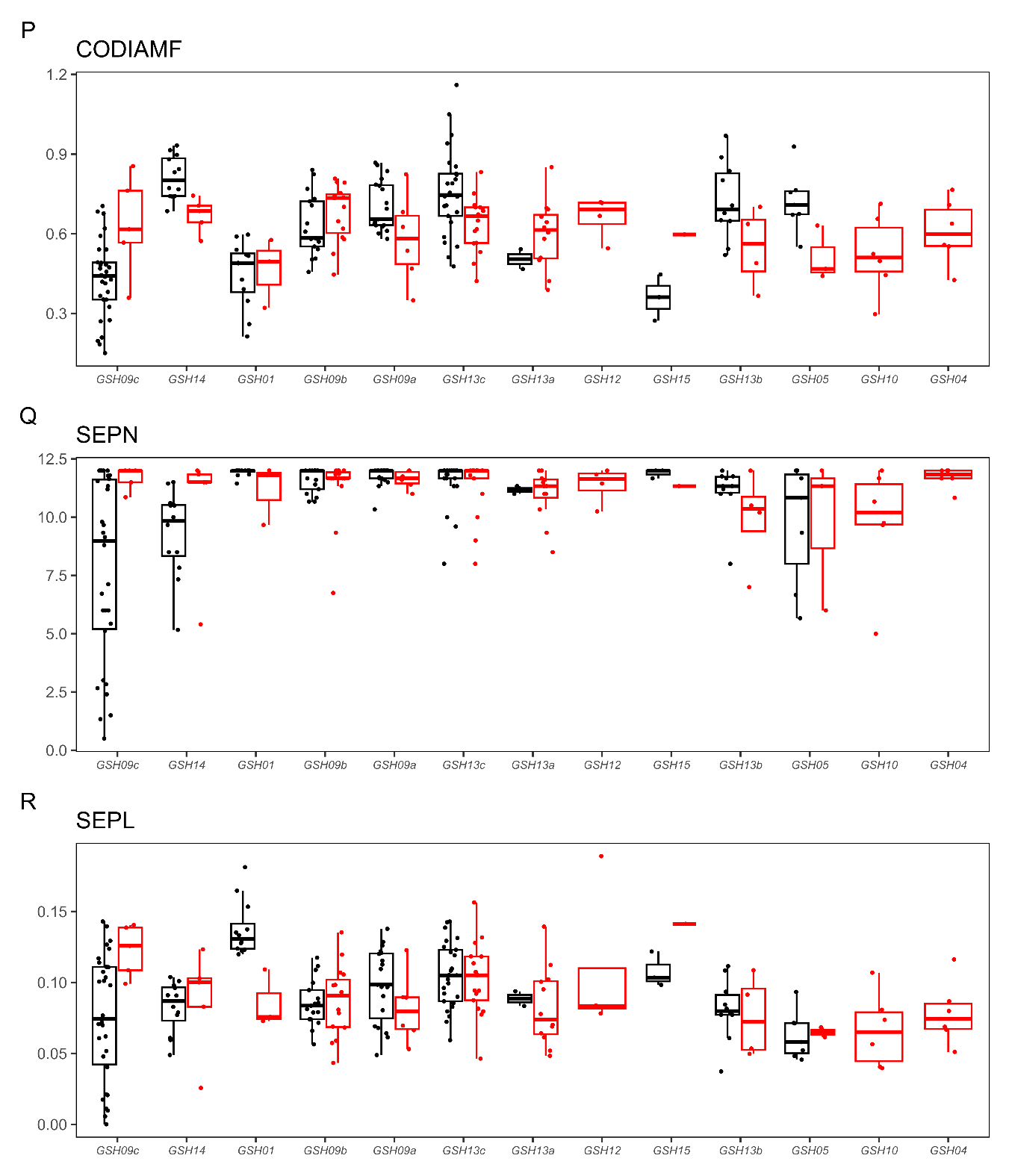


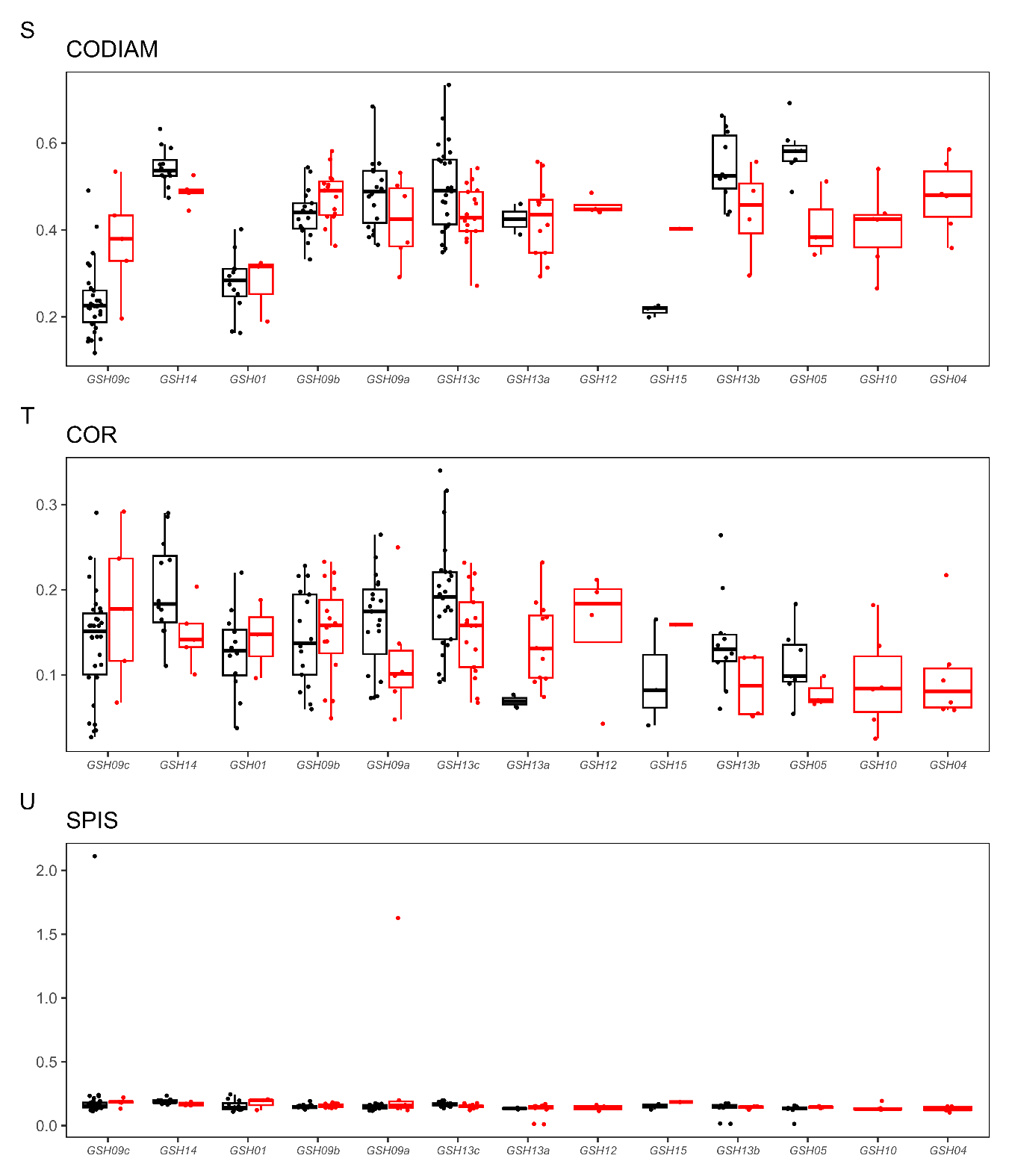


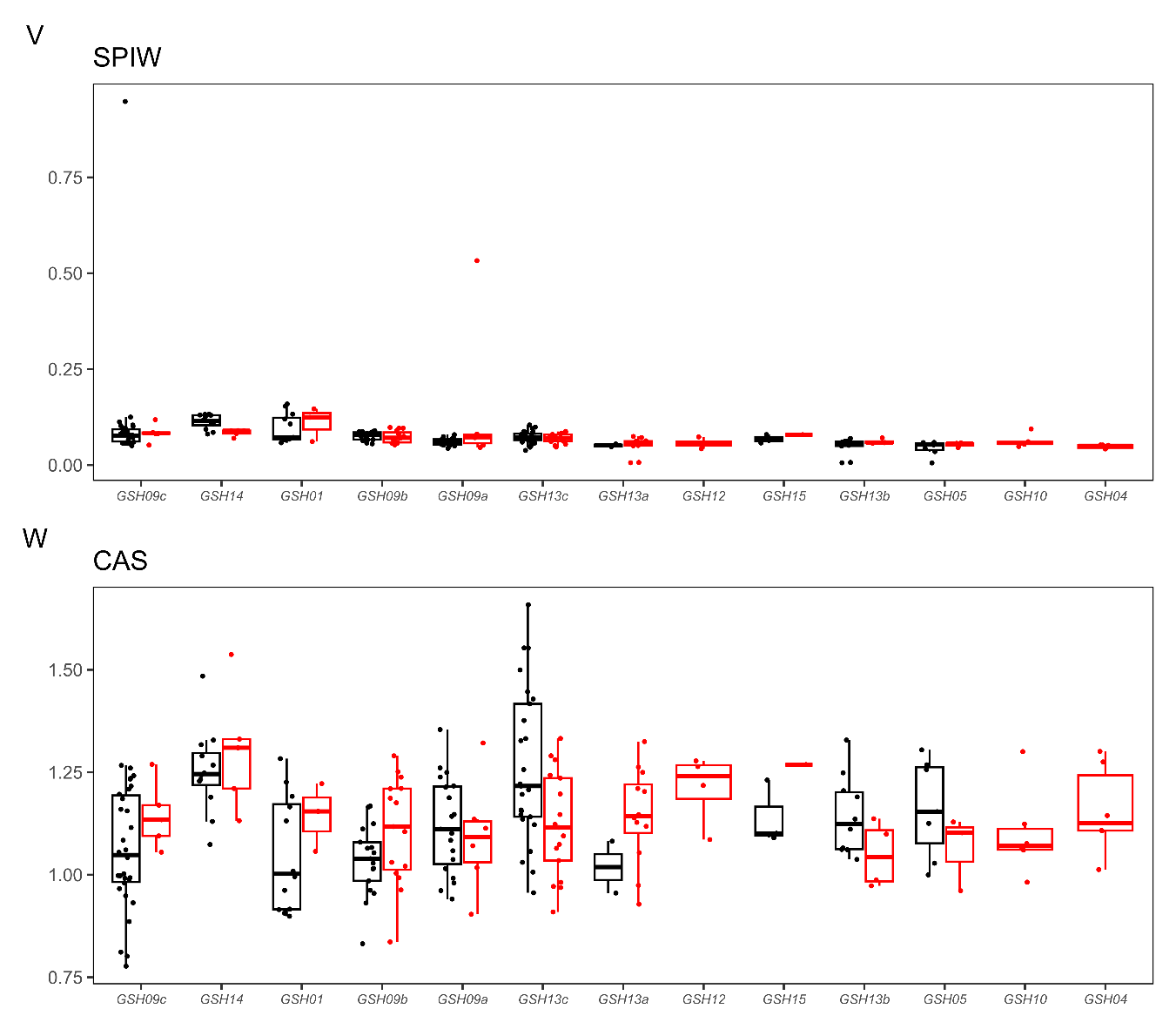


**S5 Fig**. **Univariate parameters of *Pocillopora* morphology (A-W).** Refer to Fig 2 and Table 2 for the full parameter’s description. Refer to Table S6 for each plot measurement unit. Correctly classified corals by *Pocillopora* corallum and corallite models in black, wrong classified corals in red.

## Note S1: REFERENCES FOR RESEARCH (UNCLOS) PERMITS and FOR CITES PERMITS

*UNCLOS PERMITS*

Sampling permit for PANAMA under the reference 'SE/AP-18-16' delivered by the Direccion de Areas Protegidas y Vida Silvestre - LIC. Samuel Valdez Diaz Director - Ministerio de Ambiente – Republica de Panama on the 13/06/2016; Sampling permit for PANAMA under the reference '2016-0701-2019-2' delivered by the Smithsonian Tropical Research Institute Instituto Smithsonian de Investigaciones Tropicales - STRI Animal Care and Use Committee (ACUC) on the 28/06/2016; Sampling permit for PANAMA under the reference '2016-0701-2019-2-A1' delivered by the Smithsonian Tropical Research Institute Instituto Smithsonian de Investigaciones Tropicales - STRI Animal Care and Use Committee (ACUC) on the 21/06/2018; Sampling permit for COLOMBIA under the reference 'N°009' delivered by the MINISTERIO DE AMBIENTE Y DESARROLLO SOSTENIBLEPARQUES NACIONALES NATURALES DE COLOMBIA on the 04/03/2016; Sampling permit for CHILE under the reference '13270/24/457/Vrs' delivered by the Servicio Hidrografico y Oceanografico de la Armada de Chile (SHOA) – Patricio Carrasco Hellwig Contraalmirante Director on the 29/08/2016; Sampling permit for UNITED-KINGDOM (PITCAIRN ISLANDS) under the reference 'N/A' delivered by the Government of Pitcairn islands /Environmental, Conservation & Natural Resources Division Manager // Christian Michele on the 25/02/2016; Sampling permit for COOK under the reference '11-16' delivered by the Foundation for National Research – Cook Island Research Committee – Office of the Prime Minister – Elizabeth Wright-Koteka (Chairperson) on the 12/09/2016; Sampling permit for NIUE under the reference '34/16' delivered by the Government of Niue – Office for External Affairs on the 17/11/2016; Sampling permit for SAMOA under the reference 'Memorandum of Agreement' delivered by the THE GOVERNMENT OF THE INDEPENDENT STATE OF SAMOA acting by and through the Ministry of Natural Resources and Environment on the 29/11/2016; Sampling permit for GUAM under the reference 'U2021-023' delivered by the Marine Scientific Research Coordinator Office of Ocean and Polar Affairs – United States Department of State Bureau of Oceans and International Environmental and Scientific Affairs on the 27/10/2021.

*CITES PERMITS*

CITES export permit for PANAMA (I01) under the reference 'SEX/A-72-16' delivered by the Autoridad Nacional del Ambiente (ANAM) de la República de Panamá – Autoridad Administrativa CITES on the 28/07/2016; CITES final import permit under the reference 'FR1609100066-I' delivered the 04/08/2016 by the DRIEE ILE-DE-FRANCE; CITES export permit for PANAMA (I02) under the reference 'SEX/A-72-16' delivered by the Autoridad Nacional del Ambiente (ANAM) de la República de Panamá – Autoridad Administrativa CITES on the 28/07/2016; CITES final import permit under the reference 'FR1609100066-I' delivered the 04/08/2016 by the DRIEE ILE-DE-FRANCE; CITES export permit for COLOMBIA (I03) under the reference '41499' delivered by the Ministerio de Ambiente y Desarrollo Sostenible de la República de Colombia on the 13/02/2017; CITES final import permit under the reference 'FR1707506158-I' delivered the 17/03/2017 by the DRIEE ILE-DE-FRANCE; CITES export permit for CHILE (I04) under the reference '16CL000007WS' delivered by the Servicio Nacional de Pesca y Acuicultura on the 02/09/2016; CITES final import permit under the reference 'FR1607525599-I' delivered the 03/11/2016 by the DRIEE ILE-DE-FRANCE; CITES export permit for UNITED-KINGDOM (PITCAIRN ISLANDS; I05) under the reference 'FR1698700198-E' delivered by the Haut-Commissariat de la République en Polynésie Française on the 03/11/2016; CITES final import permit under the reference 'FR1607525646-I' delivered the 04/11/2016 by the DRIEE ILE-DE-FRANCE; CITES export permit for FRENCH POLYNESIA (GAMBIER – TUAMOTU; I06) under the reference 'FR1698700198-E' delivered by the Haut-Commissariat de la République en Polynésie Française on the 03/11/2016; CITES final import permit under the reference 'FR1607525646-I' delivered the 04/11/2016 by the DRIEE ILE-DE-FRANCE; CITES export permit for MOOREA (I07) under the reference 'FR1698700218-E' delivered by the Haut-Commissariat de la République en Polynésie Française on the 21/11/2016; CITES final import permit under the reference 'FR1707503441-I ' delivered the 07/02/2017 by the DRIEE ILE-DE-FRANCE; CITES export permit for COOK (I08) under the reference 'CK/2016 – 14278' delivered by the Tu’anga Taporoporo national environment service of the Cook Islands on the 17/11/2016; CITES final import permit under the reference 'FR1707503442-I ' delivered the 07/02/2017 by the DRIEE ILE-DE-FRANCE; CITES export permit for NIUE (I09) under the reference 'N/A' delivered by the N/A on the N/A; CITES final import permit under the reference 'FR1707511900-I' delivered the 11/06/2017 by the DRIEE ILE-DE-FRANCE; CITES export permit for SAMOA (I10) under the reference 'SAMC16012' delivered by the Ministry of Natural Resources and Environment (MNRE) of the Government of Samoa on the 29/11/2016; CITES final import permit under the reference 'FR1707503440-I' delivered the 07/02/2017 by the DRIEE ILE-DE-FRANCE; CITES export permit for GUAM (I15) under the reference '17US18844C/9' delivered by the U.S. Fish and Wildlife service – Division of management authority – Branch of permits on the 02/03/2017; CITES final import permit under the reference 'FR1707503440-I' delivered the 07/02/2017 by the DRIEE ILE-DE-FRANCE.
